# Cardiac Sodium Channel Dysfunction and Dilated Cardiomyopathy: A Contemporary Reappraisal of Pathophysiological Concepts

**DOI:** 10.3390/jcm8071029

**Published:** 2019-07-12

**Authors:** Babken Asatryan

**Affiliations:** Department of Cardiology, Inselspital, Bern University Hospital, Freiburgstrasse 10, 3010 Bern, Switzerland; babken.asatryan@insel.ch; Tel.: +41-31-632-84-37; Fax: +41-31-632-42-11

**Keywords:** *SCN5A*, cardiac sodium channel, cardiac channelopathy, dilated cardiomyopathy, precision medicine

## Abstract

A key emerging theme in translational cardiovascular medicine is the need to identify specific causes of arrhythmias and heart failure, defined by phenotype and/or genotype that will respond to a particular intervention. Unlike other genes implicated in hereditary arrhythmias and cardiomyopathies, pathogenic/likely pathogenic variants in the cardiac sodium channel alpha subunit gene (*SCN5A*) produce a remarkably diverse set of electrical and structural phenotypes, one of them being dilated cardiomyopathy. There has been debate about whether left ventricular remodeling is a bona fide phenotypic feature of cardiac sodium channel dysfunction, or a consequence of tachyarrhythmias or conduction disturbances. In light of recent findings, a critical digest of the available experimental and medical literature is necessary. This paper provides a critical appraisal of the evidence linking a dysfunctional cardiac sodium channel to ventricular dysfunction, and discusses the potential mechanisms involved in shaping this phenotype along with implications for precision therapy.

## 1. Introduction

The alpha subunit of the cardiac voltage-gated sodium channel Na_V_1.5 (*SCN5A*), which is responsible for the rapid depolarization phase of the cardiac action potential, has been one of the first studied ion channels since the early days of cardiovascular genetic research [1,2,3]. Unlike other cardiac-relevant genes that are typically implicated in either cardiac channelopathies or cardiomyopathies, rare variants in *SCN5A* have been associated with an incredibly diverse spectrum of frequently overlapping electrical and structural phenotypes. Loss-of-function variants have been linked to Brugada syndrome [4], progressive cardiac conduction disease (PCCD) [5], congenital atrioventricular block [6], sick sinus syndrome [7], idiopathic ventricular fibrillation [3], and atrial standstill [8], while gain-of-function variants have been associated with long QT syndrome type 3 [1], and multifocal ectopic Purkinje-related premature contractions (MEPPC) [9]. Other *SCN5A*-mediated conditions such as familial atrial fibrillation [10], sudden infant death syndrome [11], familial dilated cardiomyopathy (DCM) [8,12], and, rarely, arrhythmogenic right ventricular cardiomyopathy (ARVC) [13], have a more complex pathophysiology with involvement of multiple molecular phenotypes. A great variation of phenotypes has been noted even within the affected families and in individual patients over time, but to date, genotype–phenotype analyses have not been able to explain this variation in a clinical phenotype.

Of more than 450 disease-causing *SCN5A* variants identified, only a handful have been linked to DCM [12]. Pathogenic/likely pathogenic variants in *SCN5A* are associated with a substantially higher burden of atrial and ventricular arrhythmias (in >90% of cases), cardiac conduction disease, and higher risk of sudden cardiac death [12,14,15,16]. While mechanisms underlying different electrical phenotypes have been profoundly studied using in vitro and in vivo approaches, little is known about how sodium channel malfunction leads to ventricular dysfunction and dilation. A critical appraisal of the existing scientific evidence might add another missing piece to this puzzle.

## 2. Clinical Evidence

**Figure 1:**
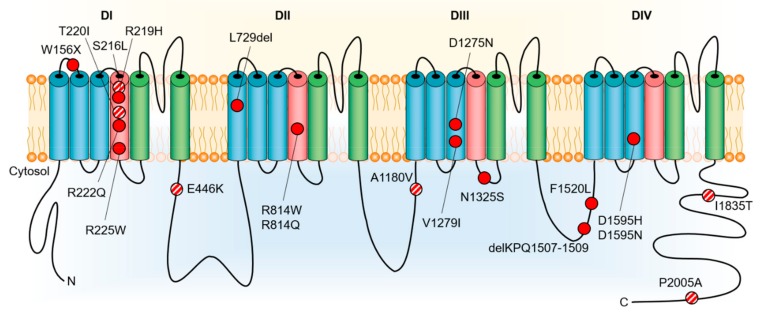
Rare variants in cardiac Na_V_1.5 voltage-gated sodium channel (*SCN5A*) reported in association with dilated cardiomyopathy. Two additional variants, c.2550-2551insTG and c.3318dupC, causing truncation of the encoded protein in patients with dilated cardiomyopathy, are not shown. Pathogenic/likely pathogenic variants are shown in red, variants with insufficient evidence of pathogenicity in dilated cardiomyopathy are shown in red/white.

Early studies reported pathogenic/likely pathogenic *SCN5A* variants in nearly 2% of all DCM cases [14]. Both sporadic cases and familial forms with autosomal dominant inheritance have been reported [12,14]. Variants associated with DCM have been localized to cytoplasmic, extracellular and transmembrane domains (DI-DIV) of Na_V_1.5 (Figure 1). The main findings that support a potential role in DCM are the familial aggregation of the trait and the segregation of *SCN5A-*variants with clinical phenotype and/or histological characteristics of DCM, with or without associated electrical abnormalities (although with reduced penetrance). A recent study, however, reported no excess variation in *SCN5A* in DCM cases versus an Exome Aggregation Consortium (ExAC) control population, suggesting that most variants in this gene are unlikely to cause DCM [17]. Instead, it has been noted that *SCN5A* is in fact one of the genes with highest background variation. Thus, establishing a causal role of an *SCN5A* variant in DCM requires very strong functional evidence of pathogenicity and/or segregation with phenotype in large pedigrees.

## 3. Experimental Evidence

Most of the reported DCM-associated *SCN5A* variants are missense variants, with a predilection for location in the S3 and S4 transmembrane domains, implicating a disruption of voltage-sensing mechanisms [15]. In vitro studies have shown that these variants commonly have loss-of-function, or infrequently gain-of-function, or rarely combined loss-of-function and gain-of-function effects on Na_V_1.5 activity [16,18,19,20,21]. Additionally, conserved variants R225P and R814W localized at the S4 of DI and DII, respectively, which were associated with an atypical phenotype combining cardiac conduction disturbances, Brugada syndrome or long QT phenotype and DCM, were shown to result in the creation of an alternative permeation pathway through the normally non-conductive voltage sensor domain (gating pore current) [22]. Yet, there is practically no in vivo or in vitro evidence directly linking *SCN5A* defects to DCM. In other words, the existing evidence on biophysical phenotypes of rare *SCN5A* variants demonstrates their potential as a substrate for arrhythmias and conduction disturbances, but does not clearly point to the pathogenesis of DCM. Digging into the thought-provoking hypotheses regarding how *SCN5A* defects might lead to ventricular dysfunction and heart failure might shed some light on this uncertainty.

## 4. DCM as a Manifestation of Pure Na_V_1.5 Dysfunction

One theory is that DCM represents a direct consequence of Na_V_1.5 channel dysfunction, meaning that the structural phenotype is primarily driven by electrical abnormalities [23,24]. Studies have suggested, that a proton leak into the cardiomyocyte through the Na_V_1.5 channel, or increased Na^+^ influx caused by gain-of-function variants, may lead to compensatory activation of the N^+^/H^+^ or the Na^+^/Ca^2+^ exchanger, thus leading to intracellular acidification or Ca^2+^ overload, respectively, and consequent impaired excitation–contraction coupling and/or myocardial damage with subsequent heart failure [22,24]. The first mechanism has been demonstrated for *SCN5A*-R219H [25], while the second was indirectly assumed for the loss-of-function A1180V missense variant [26]. These hypotheses, however, do not explain why the majority of the variants causing significant Na_V_1.5 dysfunction do not result in left ventricular (LV) dysfunction. Thus, it is more likely that mechanisms other than direct modulation of Na_V_1.5 activity are involved, such as down-regulation of channel expression, or channel mislocalization due to altered cytoskeletal anchoring [14,19]. As such, an increasing body of literature suggests interactions between sodium channel alpha subunits to form dimers [27]. Variants at the interaction sites mediating dimerization and sodium channel macromolecular complex formation might be involved in the DCM pathogenesis.

## 5. DCM Caused by Disrupted Na_V_1.5 Interaction with Partner Proteins

On the other hand, it is possible that the interaction of the defective Na_V_1.5 with its partner proteins of the cytoskeleton and intercalated disc, is responsible for the structural phenotype [28]. Interestingly, pathogenic *SCN5A* variants have been described in rare forms of left-ventricular non-compaction with a high arrhythmic burden [29]. Studies on induced pluripotent stem cell-derived cardiomyocytes have shown that the ARVC-associated missense variant *SCN5A-*R1898H leads to a significant reduction in peak *I*_Na_ current, and of the abundance of Na_V_1.5 and N-cadherin clusters at the intercalated disc [13]. Other studies have shown that *SCN5A*-positive Brugada syndrome patients have significant cardiomyopathic changes, primarily in the right ventricular outflow tract, such as fatty wall replacement, fibrosis, and reduced expression of connexin 43 [30,31,32]. Moreover, missense *PKP2* variants identified in *SCN5A-*negative Brugada syndrome patients were shown to cause a loss of expression of desmosomal protein plakophilin-2, which was associated with decreased *I*_Na_, reduced number of Na_V_1.5 channels at the intercalated disc, and increased separation of microtubules from the cell end [33]. These interactions between the cardiac sodium channel complex and the intercalated disc likely underline mechanisms relevant to *SCN5A*-medicated ARVC and *PKP2*-mediated Brugada syndrome. Could an abnormal Na_V_1.5 channel result in reduced ventricular contractility through disrupting the function of its interacting proteins and/or those already implicated in DCM, such as proteins of the sarcomere, cytoskeleton, or the dystrophin-associated glycoprotein complex?

To date, several proteins interacting with the Na_V_1.5 channel have been shown to contribute to the *SCN5A*-mediated phenotypes through their alteration of the sodium channel availability or biophysical properties. Na_V_1.5 regulatory proteins caveolin 3 (*CAV3*), alpha 1 syntrophin (*SNTA1*), and cardiac sodium channel beta subunit 4 (*SCN4B*) have been reported in association with rare subtypes of congenital long QT syndrome. Pathogenic variants in cardiac sodium channel beta subunit 3 (*SCN3B*) and glycerol 3 phosphate dehydrogenase 1-like protein have been identified in patients with Brugada syndrome. Interestingly, cardiac sodium channel beta subunit 1 (*SCN1B*) has been linked to Brugada syndrome, atrial fibrillation (also *SCN2B*), and cardiac conduction disease. A number of other proteins have been shown to interact with and regulate the Na_V_1.5 channel. These include anchoring adaptor proteins ankyrin-G, syntrophins, MOG1, plakophilin-2, enzymes, such as nedd4-like enzymes, calmodulin kinase II δc, and protein tyrosine phosphatase H1, and other proteins that modulate the channel biophysical properties, such as 14-3-3η, calmodulin, telethonin, GPD1L, and FHF1B [34]. However, strong associations between pathogenic variants in these protein genes and development of DCM have not been reported.

The sarcolemmal membrane-associated protein (SLMAP) is localized at T-tubules and sarcoplasmic reticulum. Pathogenic variants in *SLMAP* have been shown to cause Brugada syndrome via modulating the intracellular trafficking of the Na_V_1.5 channel [35]. A recent report showed that transgenic mice with cardiac-specific expression of the SLMAP isoform 3 (SLMAP3) develop a significant decrease in fractional shortening and in cardiac output without notable hypertrophy, fibrosis, or fetal gene activation [36]. Electrocardiography identified increased PR interval and a decreased R amplitude. Western blot analysis revealed a decreased protein levels of Na_V_1.5 and calcium transport system of the sarcoplasmic reticulum (SERCA2a/PLN), suggesting a selective regulatory role of SLMPA3 in ion transport proteins at the level of gene expression. It is, however, unclear whether SLMAP3 is a contributor of DCM phenotype in humans or whether any of the reported DCM-associated *SCN5A* variants disrupts an interacting domain of its partner proteins or other DCM-associated genes/proteins. The list of proteins interacting with Na_V_1.5 is also not conclusive, and many aspects require further research. It is expected that these patterns will become clearer with further experimental evidence and with more genotype-phenotype analyses on DCM and related disorders. As a first step, *SCN5A* disruption has been demonstrated to result in TGF-β_1_-mediated fibrosis in a murine model of sinus node dysfunction. It is therefore possible that *SCN5A* variants can influence the pro-fibrotic milieu associated with other protein variants, and thereby contribute to the development of DCM [37].

## 6. DCM Resulting from Long-Standing and Frequent Arrhythmias

Alternatively, high arrhythmic burden may lead to ventricular dysfunction over time. This theory was primarily based on the finding that in several cases of MEPPC and DCM due to gain-of-function *SCN5A-*R222Q variant, therapy with hydroquinidine, flecainide, or amiodarone (in addition to standard treatment of heart failure) rapidly and effectively reduced the number of multifocal premature ventricular contractions and reversed the LV remodeling [10,16,38]. Following the initial report of R222Q, similar phenotypes have been reported for R222P, I141V, and G213D [39,40,41]. Nevertheless, whether the recovery of ventricular function relates to the premature ventricular contraction burden reduction or to intracellular mechanisms secondary to pharmacological blockade of the defective sodium channel, remain to be elucidated. Furthermore, some *SCN5A*–DCM patients, such as those carrying the D1275N variant, lack a history of long-lasting ventricular arrhythmias [14], and thus the LV dysfunction in these patients is unlikely to be a consequence of ventricular arrhythmias. It is therefore more likely that this is a contributing mechanism rather than a primary cause.

## 7. DCM Secondary to Cardiac Conduction Disturbances

DCM might also develop secondary to loss-of-function variants, which leads to reduced sodium conductivity or channel availability. Classical manifestations of loss-of-function *SCN5A* variants are Brugada syndrome and PCCD (Lenège and Lev disease), but development of DCM at late disease stages, often 15 to 20 years after diagnosis, has been described in many cases. In addition, transgenic mice with 90% decreased Na_V_1.5 expression [19] and mice with the DCM-associated D1275N variant, have been found to display conduction slowing with progressive age-dependent changes suggestive for DCM [42]. This hypothesis, however, does not explain the development of DCM in rare *SCN5A* variant carriers with unaffected cardiac conduction. The majority of *SCN5A* variants causing cardiac conduction defects are frameshift/truncation variants, which produce a conduction phenotype proportionate to the severity of Na_V_1.5 dysfunction, whereas most DCM-related *SCN5A* variants are missense changes.

## 8. Mechanisms Involving Other Genetic Influences

Genome-wide association studies (GWAS) with case-control design have shown that another *SCN5A*-mediated condition, Brugada syndrome, is more likely to develop in patients who carry multiple common variants with a small effect, referred to as small nucleotide polymorphisms (SNPs). These SNPs can modulate the expression dosage of Na_V_1.5 by altering mechanisms such as the dosage of the messenger RNA, the number of channels on the surface of cardiomyocyte, or even by modulating the affinity of a transcription factor for the gene regulatory element. Considering its shared genetic substrate with *SCN5A*-mediated DCM, it is likely that the DCM is also influenced by genetic modifiers, but more GWAS need to be completed before revisiting this hypothesis. Furthermore, measurements of *I*_Na_ in HEK293 cells expressing DCM-associated variants R222Q and I1835T using whole-cell voltage clamp technique, have revealed that common polymorphisms H558R and Q1077del are relevant for their phenotypic expression and have a large impact on the Na_V_1.5 biophysical phenotypes [43]. It is therefore more likely that *SCN5A-*mediated phenotypes result from complex oligo-polygenic disease with some effect of post-translational and environmental factors, rather than a strict Mendelian inheritance.

## 9. Non-Genetic and Epigenetic Influences

The relationship between *SCN5A* variants and the risk of DCM may be influenced by factors other than genotype. Studies of a DCM family carrying the loss-of-function A1180V variant demonstrated that the defective sodium channel reduced the current conduction (manifested as QRS widening) only at high heart rates [26]. These findings speak in favor of the fact that certain *SCN5A* variants might be risk factors of DCM, and that physical activity, lifestyle, and health conditions that increase the heart rate might enhance the phenotype of carriers of certain *SCN5A* variants, such as the A1180V [26]. Alternatively, epigenetic influences on Na_V_1.5 expression might influence the phenotype development, but this hypothesis requires further investigation.

## 10. Clinical Implications

Despite the paucity of mechanistic insights into the pathogenesis of *SCN5A*-mediated DCM, one can derive clinical repercussions from the aforementioned theories. In patients with known *SCN5A* variants, a review of the literature for the description of its functional properties is necessary for selection of optimal beta-blockers, since propranolol (but not metoprolol) blocks both the peak and the late (persistent) *I*_Na_ [44]. This effect might alleviate the ventricular arrhythmias in gain-of-function *SCN5A* variant carriers, but can controversially provoke arrhythmias and cause or worsen cardiac conduction delays at different levels in those with loss-of-function variants. Likewise, the former group might also benefit from treatment with class Ic antiarrhythmic medications that block the sodium channel, such as flecainide, while its use in the latter group might elicit arrhythmias and cardiac conduction delay. These suggestions are however limited to patients with clearly pathogenic and previously studied *SCN5A* variants, and more functional studies are needed to expand our knowledge to more *SCN5A* variants. Perhaps the growing use of the automated patch clamp technique will help advance this process.

## 11. Conclusions

The aforementioned controversies suggest that neither of the living hypotheses provides an ultimate explanation according to today’s knowledge, and that molecular mechanisms responsible for Na_V_1.5/*SCN5A*-related cardiomyopathy are rather multifaceted and yet to be explored. The era of next-generation sequencing gives the advantage of identifying genetic modifiers that may play a role in shaping the DCM phenotype. However, genetic substrates alone in the absence of post-translational and/or environmental influences are unlikely to give full and conclusive explanation for this controversy. Looking ahead, the growing experience with disease modeling based on human induced pluripotent stem cell-derived cardiomyocytes and transgenic animal models, will optimistically pave the way for better characterization of Na_V_1.5 role in cellular biological processes and help identify mechanisms by which genetic and/or environmental factors affect the ventricular contractility in carriers of *SCN5A* variants. Sound evidence on disease pathogenesis will also guide us on our path for disease modification and show whether gene therapy might be a viable option for treatment of patients with *SCN5A-*mediated DCM in the near future. Before that expands our horizon, we need to adhere the conventional guidelines for management of arrhythmias and heart failure in these patients, and strictly limit our precision therapy with sodium channel blockers only as an alternative therapy to those with known gain-of-function *SCN5A* variants.
